# Comparative In Vivo Imaging of Retinal Structures in Tree Shrews, Humans, and Mice

**DOI:** 10.1523/ENEURO.0373-23.2024

**Published:** 2024-03-25

**Authors:** Marta Grannonico, David A. Miller, Mingna Liu, Michael A. Krause, Elise Savier, Alev Erisir, Peter A. Netland, Jianhua Cang, Hao F. Zhang, Xiaorong Liu

**Affiliations:** ^1^Department of Biology, University of Virginia, Charlottesville, Virginia 22904; ^2^Department of Biomedical Engineering, Northwestern University, Evanston, Illinois 60208; ^3^Departments of Ophthalmology, University of Virginia, Charlottesville, Virginia 22904; ^4^Psychology, University of Virginia, Charlottesville, Virginia 22904; ^5^Program in Fundamental Neuroscience, University of Virginia, Charlottesville, Virginia 22904

**Keywords:** in vivo imaging, mouse retina, retinal layer structure, sublayer of IPL, tree shrew, vis-OCT

## Abstract

Rodent models, such as mice and rats, are commonly used to examine retinal ganglion cell damage in eye diseases. However, as nocturnal animals, rodent retinal structures differ from primates, imposing significant limitations in studying retinal pathology. Tree shrews (*Tupaia belangeri*) are small, diurnal paraprimates that exhibit superior visual acuity and color vision compared with mice. Like humans, tree shrews have a dense retinal nerve fiber layer (RNFL) and a thick ganglion cell layer (GCL), making them a valuable model for investigating optic neuropathies. In this study, we applied high-resolution visible-light optical coherence tomography to characterize the tree shrew retinal structure in vivo and compare it with that of humans and mice. We quantitatively characterize the tree shrew's retinal layer structure in vivo, specifically examining the sublayer structures within the inner plexiform layer (IPL) for the first time. Next, we conducted a comparative analysis of retinal layer structures among tree shrews, mice, and humans. We then validated our in vivo findings in the tree shrew inner retina using ex vivo confocal microscopy. The in vivo and ex vivo analyses of the shrew retina build the foundation for future work to accurately track and quantify the retinal structural changes in the IPL, GCL, and RNFL during the development and progression of human optic diseases.

## Significance Statement

The tree shrew retina shares more similarities to human retinas than mice. In this study, we applied high-resolution visible-light optical coherence tomography to characterize the tree shrew retinal structure in vivo and compare it with that of humans and mice. We found that the tree shrew exhibits a dense retinal nerve fiber layer and thick ganglion cell layer and an inner plexiform layer (IPL). For the first time, we quantified the distinct retinal layers in tree shrew eyes, including the sublayer structures within the IPL. The analysis of the tree shrew retina, both in vivo and ex vivo, establishes a robust foundation for detecting and quantifying retinal structural changes during the development and progression of glaucoma or optic neuropathy.

## Introduction

In the retina, photoreceptors convert light into electrical signals transmitted through interneurons in the inner nuclear layer (INL) to retinal ganglion cells (RGCs). Axons of RGCs converge in the retinal nerve fiber layer (RNFL) to the optic nerve head (ONH) and then form the optic nerve, which conveys the information to the higher visual centers in the brain (Cajal, 1972; Sernagor et al., 2001; Sanes and Masland, 2015; Seabrook et al., 2017; Cang et al., 2018). In diseased conditions such as glaucoma or optic neuropathy, RGCs may have compartmentalized self-destructive programs, which result in dendritic changes in the inner plexiform layer (IPL), soma degeneration in the ganglion cell layer (GCL), and axon degeneration in the RNFL (Whitmore et al., 2005; Puyang et al., 2015; Quigley, 2016; Tatham and Medeiros, 2017; Syc-Mazurek and Libby, 2019; Grannonico et al., 2023). For example, studies suggest that RGC dendrite degeneration may precede detectable soma abnormalities in glaucoma (Feng et al., 2013b; Chen et al., 2015; El-Danaf and Huberman, 2015; Ghassabi et al., 2022). Therefore, in vivo visualization and quantification of the structural changes in the IPL, GCL, and RNFL may provide valuable indicators for RGC damage. However, the optimal diagnosis and management of ocular diseases remains challenging, which depends on disease progression (Hood et al., 2007, 2009; Hood, 2017), resolution of the imaging systems (Tatham and Medeiros, 2017; Hou et al., 2018; Schuman et al., 2020; Liu and Zhang, 2023), and sensitivity to detect the layer thinning due to the RGC damage (Quigley, 2016; Grannonico et al., 2023).

Rodent models of glaucoma have been widely used (Aihara et al., 2003; Grozdanic et al., 2003; Mabuchi et al., 2003; Feng et al., 2013a; Liu et al., 2020) because they share basic retinal structures with the human retina and offer genetic tools for in vivo manipulations (John, 2005; Howell et al., 2008). However, rodents have small eyes with thin layers, which impose challenges for investigating structural changes in retinal diseases. Notably, rodents are mostly nocturnal mammals (Szél and Röhlich, 1992; Jeon et al., 1998), and their total number of RGCs is <10% compared with primate retinas (Cull et al., 2003), reflecting a more primitive visual system. In contrast, tree shrews (*Tupaia belangeri*) are diurnal mammals with cone-dominant retinas and highly developed visual pathways and primary visual cortex (Lund et al., 1985; Müller and Peichl, 1989; Sajdak et al., 2019). Tree shrews are also closely related to primates and humans (Campbell, 1966), sharing many similar features to human eyes, including a fovea-like structure (Albon et al., 2007; Sajdak et al., 2019). Therefore, tree shrew retinas may represent an ideal model for studying optic neuropathies that will complement rodent studies.

To continue the development of the tree shrew as an animal model, here we provide a comparative analysis of the retinal layers in mice, tree shrews, and humans. We used visible-light optical coherence tomography (vis-OCT) to acquire high-resolution in vivo retinal images from mice, tree shrews, and humans. For the first time, we visualized and quantified the IPL sublamination in tree shrew eyes in vivo. Furthermore, we examined the similarities and differences among the RNFL, GCL, INL, and outer nuclear layer (ONL) in tree shrews, mice, and humans.

## Materials and Methods

### Subject recruiting

This study was approved by the Institutional Review Board (IRB) and Health Sciences Research (HSR) of the University of Virginia (IRB00000447, HSR210376). Three eyes from three healthy adult subjects were imaged (subject 1: Caucasian female 28 years old, right eye; subject 2: Caucasian female 32 years old, left eye; and subject 3: Caucasian male 30 years old, left eye), with no preference for left or right eye. Informed consent was obtained from each subject before imaging. Inclusion criteria included normal eyes with the spherical equivalent refractive error between −4.00 and +4.00 D sphere and best-corrected visual acuity of 20/40 or better, intraocular pressure ≤21 mmHg, normal appearance of the ONH and RNFL, cup-to-disc ratio difference <0.2 in both vertical and horizontal dimensions, and no prior history of intraocular surgery. Exclusion criteria include a history of existing retinal pathologies, significant ocular media opacity, any evidence of vitreoretinal or macular disease, optic neuropathy, ocular trauma, or diabetes.

### Animal preparation

All tree shrew and mouse protocols were approved by the University of Virginia Institutional Animal Care and Use Committee and complied with the National Institutes of Health guidelines. Healthy 2–6-month-old wild-type C57BL/6 mice and 9–24-month-old northern tree shrews (*T. belangeri*) of either sex were used. Mice were anesthetized as previously described (Miller et al., 2020; Grannonico et al., 2021). Tree shrews were initially anesthetized using 5% isoflurane with supplemental oxygen at a flow rate of 1 L/min followed by an intraperitoneal cocktail injection of ketamine (100 mg/kg; Henry Schein Medical Animal Health) and xylazine (20 mg/kg; Akorn). Tropicamide eye drops (1%; Henry Schein Animal Health) and phenylephrine (2.5%; Henry Schein Animal Health) were given to dilate the pupil and induce cycloplegia. During imaging, animals were kept warm using an infrared heat lamp, and polyvinyl alcohol artificial tears (1.4%; Rugby Laboratories) were applied between image acquisitions to prevent corneal dehydration. After imaging, animals were placed on a heating pad and monitored until alert and active.

### Vis-OCT

Adult healthy subjects were imaged with Aurora X2 vis-OCT system (Opticent Health). The Aurora X2 offers an axial resolution of 1.3 µm in the retina, a 40 kHz A-line rate, and a lateral resolution of 7.0 µm at the center of the field of view (FOV) and 12.0 µm in the peripheral FOV. A single vis-OCT volume consists of 512 A-lines/B-scan × 512 B-scans, acquired in 7.6 s. The scan covered a 3 × 3 × 1.5 mm^3^ in the retina. Based on the American National Standards Institute (ANSI) safety guidelines, the maximum permissible exposure to visible light with a 560 nm center wavelength at the pupil for a human is 5.1 J/cm^2^ (Delori et al. 2007; Yi et al., 2015; Chong et al., 2018). Therefore, for a 3 mm × 3 mm scan area on the human retina, the maximum permissible exposure for a 7.6 s scan is 60 mW. To guarantee safety, we limited the power to 250 μW, which allows ∼30 min of continuous scanning before exceeding the maximum permissible exposure. Before each imaging session, vis-OCT irradiation power was measured using a calibrated power meter (PM100D; Thorlabs; Ghassabi et al., 2022). We adjusted the optical focus and the reference arm path length for each FOV to maximize image quality. For each subject, we acquired two vis-OCT volumes from the same eye: one with the fovea aligned in the center of the FOV and the other with the ONH aligned in the center of the FOV.

Mice and tree shrews were imaged with a small animal vis-OCT system, Halo 100 (Opticent Health), as previously reported (Miller et al., 2020; Grannonico et al., 2021, 2023). The system used broadband visible light from 510 to 610 nm with an incident power on the cornea of 1 mW. The system was scanned with an A-line rate of 75 kHz with an integration time of 12.6 μs/A-line. Halo 100 offers a 1.3 μm axial resolution in the retina for both mice and tree shrews (Miller et al., 2020; Grannonico et al., 2021). Due to the differences in eye size between mice and shrews, the focus of the system was adjusted to accommodate the refractive power. Before image acquisition, eyes were aligned to maximize retinal reflectance by minimizing retinal curvature throughout the volume. For mice, the lateral resolution was 4.5 µm at the center of the FOV, and 8.7 µm at 350 µm from the center (Miller et al., 2020; Grannonico et al., 2021), and for shrews, 4.5 µm at the center of the FOV, and 8.7 µm at 560 µm from the center (Norton and McBrien, 1992; Miller et al., 2020). Isometric vis-OCT volumes consisting of 512 A-lines/B-scan × 512 B-scans/volume (with each B-scan repeated twice) were acquired, capturing a total volume of 0.7 mm × 0.7 mm × 1.5 mm (*x* × *y* × *z*) in mice and 1.12 mm × 1.12 mm × 1.5 mm (*x* × *y* × *z*) in tree shrews. A single acquisition needs ∼7 s to complete.

### Vis-OCT volume processing

Each vis-OCT volume acquisition was digitally resampled to generate speckle-reduced (SR) B-scans (SR-B-scans) at different radii (Grannonico et al., 2021). To do so, we manually marked the ONH in the enface image and plotted a ∼15-μm-thick arc around the ONH at 180 μm (central) and 500 μm (peripheral) radii in the mouse retina; at 500 μm (central) and 1,200 μm (peripheral) radii in the tree shrew retina; and 1,500 μm (central) and 4,000 μm (peripheral) radii in the human retina. Adjacent A-lines within a 0.1° sector were averaged to reduce speckle noise while preserving spatial density. Central and peripheral B-scan locations in mouse, tree shrew, and human retinas were chosen based on their eye size to keep the same distance ratio.

### Quantification of retinal layers

We measured the retinal layer thickness using MATLAB. The retinal layer thickness was recorded by extracting the axial intensity profile of the retinal layers and recording the thickness value at 1/*e*^2^. Regions under blood vessels were excluded from analysis by identifying the dark shadows caused by the attenuation of blood in vis-OCT B-scan images. Briefly, the overall retinal thickness was measured as the distance between the vitreous/RNFL and retinal pigment epithelium (RPE)/choroid boundary. The thickness of the RNFL was between the bottom edge of the internal limiting membrane and the top edge of the GCL. The thickness of the GCL was between the bottom edge of the RNFL and the top edge of the IPL, and the thickness of the IPL was between the bottom of the GCL and the top of the INL. The INL was measured between the bottom of the IPL and the top of the OPL, and the ONL was measured between the bottom edge of the OPL and the top edge of the external limiting membrane. To keep the measurement technique consistent for all structures, we sampled the retinal layer thickness measurements at evenly spaced intervals (∼150 μm) for each retina.

IPL sublayers were measured in tree shrews by resampling SR-B-scans at 500 μm (central) and 1,200 μm (peripheral) radii from the ONH. To measure the variation in the IPL sublaminal structure, we extracted SR-A-lines by registering and averaging 250 adjacent A-lines from the resampled B-scan images. In the averaged A-line profile, we identified two main peaks and one main valley corresponding to the high-intensity and low-intensity bands of contrast signal in the SR-B-scans. From the averaged A-line profiles, we manually measured the thickness of individual IPL sublamina as the number of pixels. Specifically, sublayer S_1_ was measured from the top IPL boundary to the bottom of the first peak; sublayer S_2_ was measured from the bottom of the first peak to the top of the main valley; and sublayer S_3_ was measured from the top of the main valley to the bottom IPL boundary.

### Immunohistochemistry and confocal microscopy

After acquiring vis-OCT data, mice and tree shrews were killed with Euthasol (3.9 mg/ml pentobarbital, 0.5 mg/ml phenytoin sodium; Virbac ANADA, no. 200-071) and perfused with 4% paraformaldehyde (PFA; ChemCruz, sc-281692). Mouse and three shrew eyes were immunostained using the same protocol (Gao et al., 2021; Grannonico et al., 2021; Chang et al., 2023). Briefly, eyecups were dissected and fixed in PFA for 30 min. For cryosection samples, the eye cups were cryoprotected in 30% sucrose solution overnight, embedded in an optimal cutting temperature medium (Sakura Finetek), and sectioned at 20–30 μm on a cryostat (Leica, CM1950). Sections containing the ONH area were used for this study.

For whole-mount samples, retinas were marked on the temporal side to indicate orientation. Retinas were washed with phosphate-buffered saline containing Triton X detergent (0.5% Triton X-100) and then blocked for 2 h at room temperature in a blocking buffer (2.5% BSA and 5% normal donkey serum, 0.5% Triton X-100; Sigma-Aldrich). Samples were incubated with a primary antibody overnight at 4°C. A full list of primary antibodies that we tested in tree shrew and mouse retinas are summarized in Table 1. Primary antibodies used in this study are highlighted with ^a^ in Table 1. Samples were then incubated with a secondary antibody (1:1,000) overnight at 4°C. The secondary antibodies are summarized in Table 2.

**Table 1.** A list of the primary antibodies tested in tree shew and mouse retinas

Primary antibody	Full name	Manufacturer catalog #	RRID	Host species	[C]	Works on *TS/MS*
^a^AP2-α	Activating protein-2	DSHB catalog #3B5	AB_2313947	Mouse	1:200	Y/Y
β-III tubulin-488		BioLegend catalog #801203	AB_2564757	Mouse	1:500	Y/Y
β-III tubulin		Sigma-Aldrich catalog #AB9354	AB_570918	Chicken	1:200	Y/Y
β-III tubulin		Abcam catalog #AB52623	AB_869991	Rabbit	1:200	Y/Y
^a^Brn-3a	Brain-specific homeobox-3a	Millipore catalog #MAB1585	AB_94166	Mouse	1:200	Y/Y
^a^Calbindin		Abcam catalog #ab108404	AB_10861236	Rabbit	1:200	Y/Y
Calbindin		Sigma-Aldrich catalog #C9848	AB_476894	Mouse	1:500	Y/Y
Calretinin		Abnova catalog #MAB5054	AB_10632663	Rabbit	1:500	N/Y
CD31	Cluster differentiation 31	BD Biosciences catalog #550274	AB_393571	Rat	1:50	N/Y
CD31	Cluster differentiation 31	Gift from Dr. Ribic, UVA		Mouse	1:200	N/Y
CD34	Cluster differentiation 34	Abcam catalog #ab81289	AB_1640331	Rabbit	1:1,000	N/Y
CD31	Cluster differentiation 31	Abcam catalog #ab28364	AB_726362	Rabbit	1:50	N/Y
CD144	Cluster differentiation 144	Thermo Fisher Scientific Cat#14-1441-82	AB_842767	Rat	1:200	N/Y
^a^ChAT	Choline acetyltransferase	Thermo Fisher Scientific catalog #PA5-29653	AB_2547128	Rabbit	1:500	Y/Y
^a^GAD67	Glutamate decarboxylase 67	Millipore catalog #MAB5406	AB_2278725	Mouse	1:250	Y/Y
^a^GFAP	Glial fibrillary acidic protein	Abcam catalog #ab4674	AB_304558	Chicken	1:200	Y/Y
GFAP	Glial fibrillary acidic protein	Synaptic Systems catalog #173002	AB_887720	Rabbit	1:500	Y/Y
Iba-1	Ionized calcium-binding adaptor-1	Abcam catalog #ab5076	AB_2224402	Goat	1:500	Y/Y
Icam-II	Intercellular adhesion molecule-II	BD Biosciences catalog #553325	AB_394783	Rat	1:500	N/Y
Melanopsin		Gift from Dr. Provencio, UVA		Rabbit	1:2,000	N/Y
NFH	Neurofilament heavy chain	Bio-Rad catalog #MCA1321GA	AB_1102789	Rat	1:200	N/Y
PKC-α	Protein kinase C	Thermo Fisher Scientific catalog #MA1-157	AB_2536865	Mouse	1:50	N/Y
Prox1	Prospero homeobox protein-1	R&D Systems catalog #AF2727	AB_2170716	Goat	1:250	N/Y
PV	Parvalbumin	Synaptic Systems catalog #195002	AB_2156474	Rabbit	1:500	N/N
^a^RBPMS	RNA-binding protein multiple splicing	Abcam catalog #ab152101	AB_2923082	Rabbit	1:250	Y/Y
RBPMS	RNA-binding protein multiple splicing	Abcam catalog #ab194213,	AB_2920590	Rabbit	1:200	N/Y
SMI32-488	Neurofilament H nonphosphorylated	BioLegend catalog #801705	AB_2715876	Mouse	1:500	N/Y
^a^TH	Tyrosine hydroxylase	Millipore catalog #MAB318	AB_2201528	Mouse	1:200	Y/Y
^a^Tuj1	class III β-tubulin	Gift from Dr. Spano, UVA		Mouse	1:200	Y/Y
^a^VGLUT1	Vesicular glutamate transporter 1	Synaptic Systems catalog #135302	AB_887877	Rabbit	1:500	Y/Y
VWF	Von Willebrand factor	Millipore catalog #AB7356	AB_92216	Rabbit	1:250	N/Y
VWF	Von Willebrand factor	Thermo Fisher Scientific Cat#PA5-16634	AB_10982615	Rabbit	1:100	N/Y

[C], concentration; *TS*, tree shrew; *MS*, mouse; Y, yes; N, no.

^a^ Antibodies used in this study.

**Table 2.** A list of the secondary antibodies used in tree shrew and mouse retinas

Secondary antibody	Manufacturer catalog #	RRID	Concentration
Donkey anti-mouse 488	Thermo Fisher Scientific catalog #A-21202	AB_141607	1:1,000
Donkey anti-mouse 594	Thermo Fisher Scientific catalog #A-21203	AB_141633	1:1,000
Donkey anti-mouse 647	Thermo Fisher Scientific catalog #A-31571	AB_162542	1:1,000
Donkey anti-rabbit 488	Thermo Fisher Scientific catalog #A-21206	AB_2535792	1:1,000
Donkey anti-rabbit 594	Thermo Fisher Scientific catalog #A-21207	AB_141637	1:1,000
Donkey anti-rabbit 647	Thermo Fisher Scientific catalog #A-31573	AB_2536183	1:1,000
Donkey anti-chicken 488	Sigma-Aldrich catalog #SAB4600031	AB_2721061	1:1,000
Donkey anti-goat 568	Thermo Fisher Scientific catalog #A-11057	AB_2534104	1:1,000

After immunostaining, whole-mount retinas were flat mounted and cut into four quadrants: temporal (T), nasal (N), inferior (I), and superior (S). The samples were then coverslipped with a VECTASHIELD mounting medium (Vector Laboratories; Feng et al., 2013b; Miller et al., 2020, 2023; Grannonico et al., 2021, 2023). Confocal microscopy was performed using the 3D Z-stack mode on a Zeiss microscope (LSM 800, Carl Zeiss AG; Miller et al., 2020; Thomson et al., 2020; Grannonico et al., 2021). Whole retina pictures were captured using the tiling/stitch function in Zen at 10× magnification and 1.24 µm/pixel. Cross-sectional images were taken in proximity to the ONH region (500 µm range lateral distance per mouse and 1,000 µm per tree shrew) as individual Z-stack images using a magnification of 20× and 0.64 µm/pixel. Z-stack slices were then projected to create 2-D en face confocal images using Zeiss Zen or Imaris (Imaris 9.6, Bitplane; Miller et al., 2020; Thomson et al., 2020; Grannonico et al., 2021).

### Statistical analysis

One-way analysis of variance (ANOVA) test was used to compare the image quality metrics and retinal layer thickness among multiple groups. Comparisons between the two groups were performed using a post hoc Tukey’s test with *, *p* < 0.05; **, *p* < 0.01; ***, *p* < 0.001; and ****, *p* < 0.0001. All results were reported as mean ± standard deviation.

## Results

### Vis-OCT imaging of mouse, tree shrew, and human retinas

**Figure 1.**
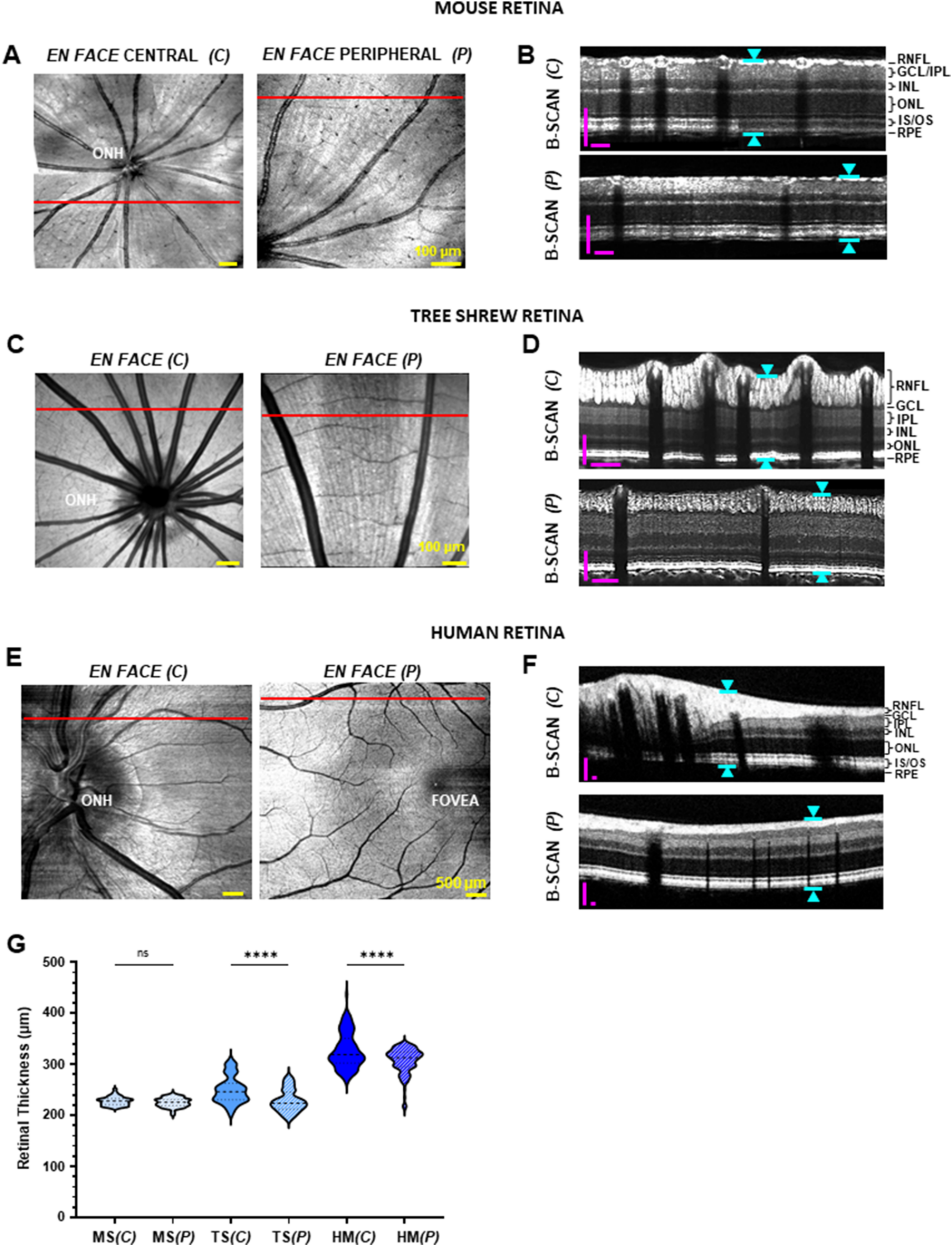
In vivo resampled SR-B-scan images of mice, tree shrews, and humans. ***A***, ***C***, ***E***, En face projection of central and peripheral retinas from a mouse (***A***), tree shrew (***C***), and human (***E***). ***B***, Resampled SR-B-scan images in mouse reconstructed along red line draw in (***A***) within 180 μm (central) and 500 μm (peripheral) distance from the ONH. ***D***, Resampled SR-B-scan images in the three shrew reconstructed along red line draw in (***C***) within 500 μm (central) and 1,200 μm (peripheral) distance from the ONH. ***F***, Resampled SR-B-scan images in a healthy volunteer reconstructed along red line draw in (***E***) centered on the ONH with 1,500 μm (central) and 4,000 μm (peripheral) radii. Blue arrows in ***B***, ***D***, and ***F*** indicate the overall retinal thickness. ***G***, Retinal thickness measurements recorded along the orthogonal trajectories (*n* = 4 mice; *n* = 3 tree shrews; *n* = 3 adult healthy volunteers). ONH, optic nerve head; RNFL, retinal nerve fiber layer; GCL, ganglion cell layer; IPL, inner plexiform layer; INL, inner nuclear layer; ONL, outer nuclear layer; IS/OS, inner/outer photoreceptor segment; RPE, retinal pigment epithelium. MS, mice; TS, tree shrews; HS, human. (*C*), central; (*P*), peripheral. Magenta scale bar, 100 µm. ns, not significant; ****p < 0.0001. One-way ANOVA post hoc Tukey’s tests, same for all figures.

With improved axial resolution achieved by vis-OCT, we were able to compare the in vivo retinal layer structures in mice, tree shrews, and humans. En face projections in the central (*C*) and peripheral (*P*) regions of mouse, tree shrew, and human retinas are shown in Figure 1*A*,*C*, and *E*, respectively, where the red lines indicate the paths along which the B-scans were reconstructed. SR-B-scans in Figure 1*B*,*D*, and *F* revealed the different morphologies among mouse, tree shrew, and human retinas, respectively. The RNFL structure in mice was largely similar in the central and peripheral SR-B-scans (Fig. 1*B*), suggesting a more homogeneous cross-sectional layer organization throughout the retina. By contrast, RGC axon bundles in the tree shrew RNFL appeared to be vertically elongated, densely packed, and stratified in the central area, while in the peripheral area, they formed a monolayer of round or oval-shaped axon bundles (Fig. 1*D*), as previously identified (Miller et al., 2023). B-scans in humans also revealed a relatively thick RNFL in the central area and a thin and linear RNFL around the fovea region (Fig. 1*F*).

We measured the overall retinal thickness from the resampled SR-B-scans at different locations and plotted the measurements in Figure 1*G*. The human central retina was significantly thicker compared with the peripheral retina (*C*, 327.1 ± 34 µm; *P*, 306.9 ± 25 µm; *n* = 120 measurements from three eyes; *p* = 1.0 × 10^−4^). In the tree shrew, the central retina was found to be 9% thicker compared with the peripheral retina (*C*, 250.7 ± 25 µm; *P*, 229.8 ± 21 µm; *n* = 88 measurements from three eyes; *p* = 1.0 × 10^−4^). There was no significant thickness change between the central and peripheral regions in the mouse retinas (*C*, 227.4 ± 8.5 µm; *P*, 224.8 ± 8.6 µm; *n* = 88 measurements from four eyes; *p* = 0.98; ANOVA post hoc Tukey’s tests; same below).

### Quantification of retinal layer thickness in mouse, tree shrew, and human

**Figure 2.**
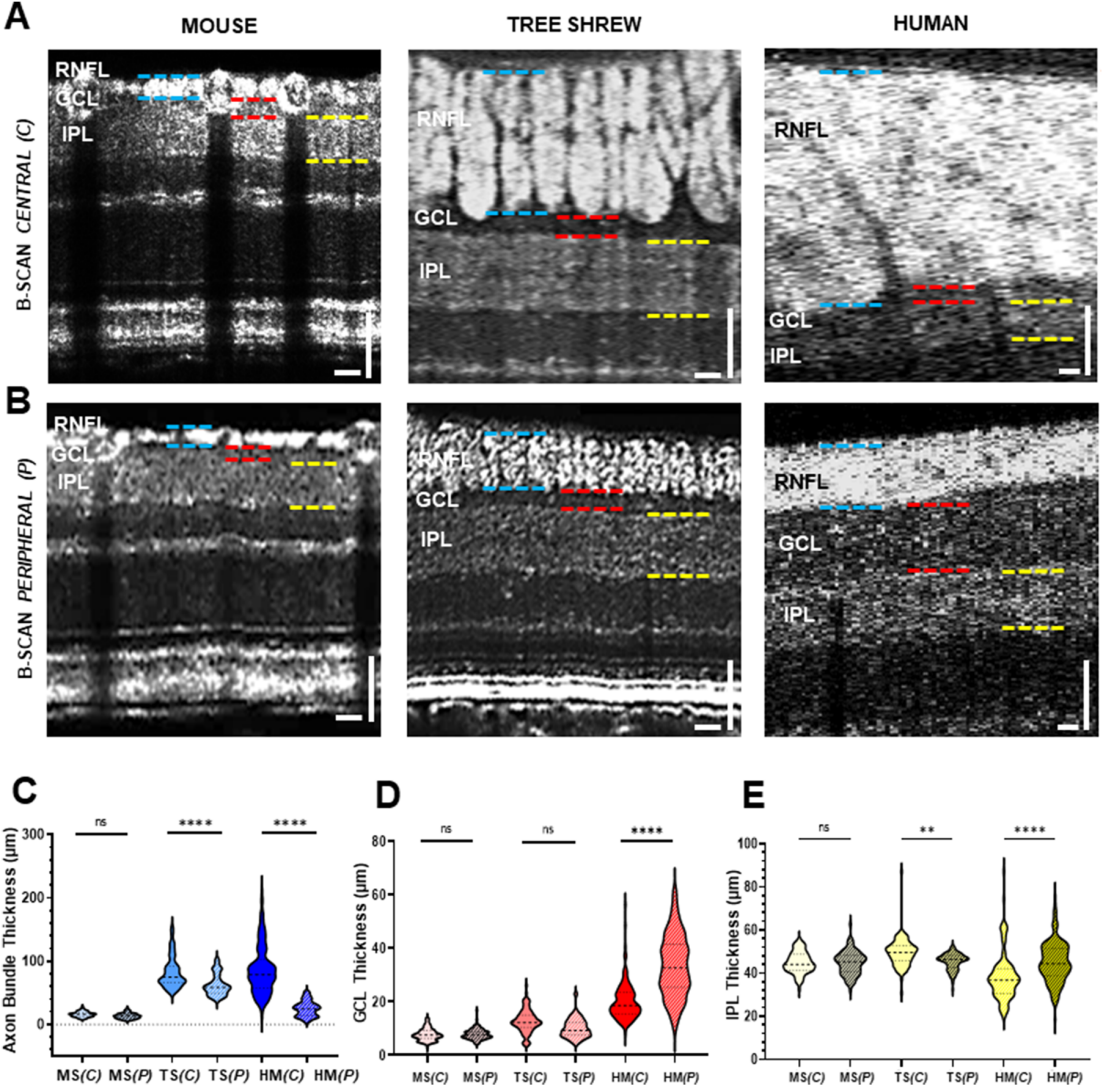
In vivo SR-B-scan comparison in the mouse, tree shrew, and human. ***A***, ***B***, SR-B-scan images from central (***A***) and peripheral (***B***) retinas showing the RNFL, GCL, and IPL in the mouse (left panel), tree shrew (middle panel), and human (right panel). Blue dashed lines highlight the RNFL boundaries, red dashed lines highlight the GCL boundaries, and yellow dashed lines highlight the IPL boundaries. ***C–E***, Quantifications of the axon bundle height measurements (***C***), GCL thickness measurements (***D***), and IPL thickness measurements (***E***) from central and peripheral SR-B-scan images in the mouse (*n* = 4), tree shrew (*n* = 3), and healthy volunteers (*n* = 3). RNFL, retinal nerve fiber layer; GCL, ganglion cell layer; IPL, inner plexiform layer; MS, mice; TS, tree shrews; HS, human. (*C*), central; (*P*), peripheral. White scale bar, 50 µm. ns, not significant; **p < 0.01; ****p < 0.0001.

To examine the variation in the retinal layer structure in mice, tree shrews, and humans, we quantified the thickness of the RNFL, GCL, IPL, INL, and ONL in vivo. Examples of the SR-B-scans captured in the central and peripheral retinas in mice, tree shrews, and humans are shown in Figure 2*A* and *B*. Mice exhibited a monolayer of round axon bundles (blue dashed lines) followed by less clear edges of the GCL (red dashed lines) and the IPL (yellow dashed lines) in both peripheral and central regions. However, tree shrews presented vertically elongated and densely packed axon bundles (blue dashed lines) in the central region compared with a more spread-out axon bundle organization toward the peripheral region with a distinct dark band of the GCL (red dashed lines) and a clear sublayered IPL band in both central and peripheral retinas (Fig. 2*A*,*B*, middle panel, yellow dashed lines). Similar to tree shrew eyes, SR-B-scans in human eyes resolved a thick and densely packed RNFL (blue dashed lines) in the central region, a thin RNFL in the peripheral region, a thick dark band of the GCL (red dashed lines) in the peripheral region, and a bright band of the IPL in both central and peripheral retinas (Fig. 2*A*,*B*, left panel, yellow dashed lines).

The in vivo quantification of the axon bundle height showed no difference in mouse between central and peripheral retinas (*C*, 16.0 ± 4.3 µm; *P*, 13.4 ± 4.2 µm; *n* = 88 measurements from four different eyes; *p* = 0.97). In contrast, significantly higher axon bundles were found in the central retina of the three shrew compared with those in the peripheral retina (*C*, 81.7 ± 23 µm; *P*, 62.8 ± 17 µm; *n* = 88 measurements from three different eyes; *p* = 1.0 × 10^−4^), coherently with the axon bundle height variation across the tree shrew retina as previously reported (Miller et al., 2023). In human eyes, the axon bundle height in the central area was found to be four times higher compared with the peripheral area (*C*, 86.3 ± 38 µm; *P*, 24.9 ± 12 µm, *n* = 120 measurements from three different eyes; *p* = 1.0 × 10^−4^; Fig. 2*C*). Overall, the axon bundle height decreased by 23% in tree shrews and by 71% in humans as distance increased from the ONH.

Figure 2*D* shows the in vivo quantification of the GCL thickness. There was no significant difference in the GCL thickness between the central and the peripheral retinas in mice (*C*, 7.7 ± 2.2 µm; *P*, 7.9 ± 2.0 µm; *n* = 88 measurements from four eyes; *p* = 0.99) as well as in tree shrews (*C*, 12.5 ± 4.5 µm; *P*, 10.1 ± 3.7 µm; *n* = 88 measurements from four eyes; *p* = 0.99). However, human retinas had a significantly thinner GCL close to the ONH (*C*, 20.2 ± 7.2 µm; *P*, 33.4.8 ± 11 µm; *n* = 120 measurements from three eyes; *p* = 1.0 × 10^−4^).

Next, we assessed the thickness of the IPL in vivo. In the mouse image, the IPL appears merged with the GCL, forming a single layer (Ruggeri et al., 2007; Huber et al., 2009; Ferguson et al., 2012; Srinivasan et al., 2014; Dysli et al., 2015), where the top edge of the IPL is outlined by a bright, thin band (Fig. 2*A***,***B*, left panel, yellow dashed lines). In the tree shrew image, the detected IPL boundaries consisted of two bright strata divided by a dark band in the middle (Fig. 2*A*,*B*, middle panel, yellow dashed lines). In the human image, the IPL was detected as a bright band right under the GCL (Fig. 2*A*,*B*, right panel, yellow dashed lines). The recorded IPL thickness from central and peripheral SR-B-scan images is plotted in Figure 2*E*. In mice, the average IPL thickness was 44.9 ± 4.9 µm in the central region and 44.9 ± 5.4 µm in the peripheral region (*p* = 0.99). In tree shrews, the average IPL thickness was 50.0 ± 5.9 µm in the central region and 45.7 ± 3.4 µm in the peripheral region (*p* = 5.1 × 10^−3^). In human eyes, the IPL thickness recorded was 38.2 ± 11 µm in the central region and 45.0 ± 9.6 µm in the peripheral region (*p* = 5.1 × 10^−3^). As distance increased from the ONH, the IPL thickness decreased by 9% in tree shrews and increased by 17% in human eyes (Beckmann et al., 2021).

**Figure 3.**
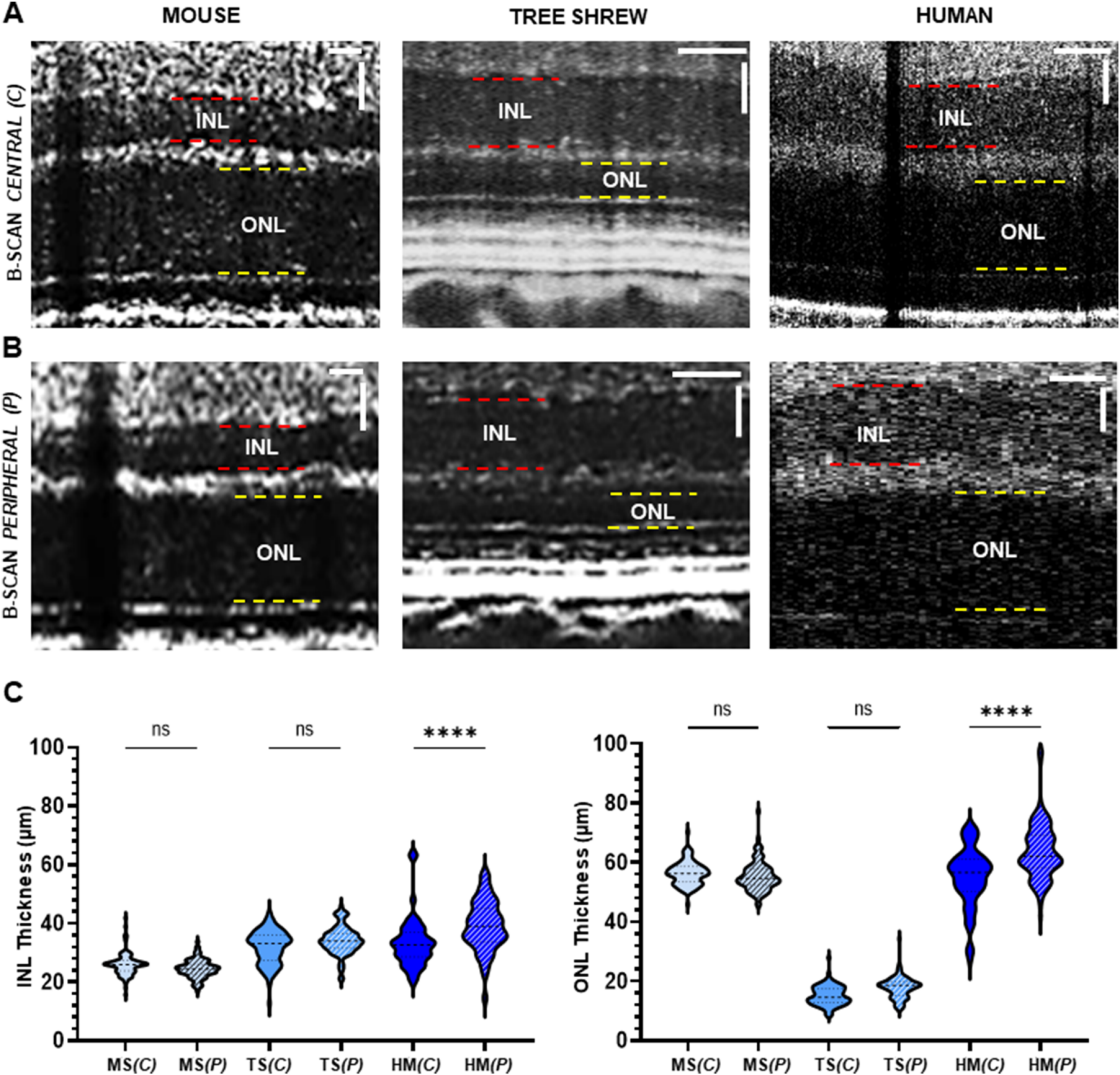
In vivo measurements of the INL and ONL thickness in the mouse, tree shrew, and human. ***A***, ***B***, SR-B-scan images from central (***A***) and peripheral (***B***) retinas showing the INL and ONL in the mouse (left panel), tree shrew (middle panel), and human (right panel). Red dashed lines highlight the INL boundaries, and yellow dashed lines highlight the ONL boundaries. ***C***, INL (left graph) and ONL (right graph) thickness measurements from central and peripheral SR-B-scan images in the mouse (*n* = 4), tree shrew (*n* = 3), and healthy volunteers (*n* = 3). INL, inner nuclear layer; ONL, outer nuclear layer. MS, mice; TS, tree shrews; HS, human. (*C*), central; (*P*), peripheral. White scale bar, 25 µm. ns, not significant; ****p < 0.0001.

The thickness of the INL and ONL were assessed in mouse, tree shrew, and human retinas. Vis-OCT SR-B-scan images revealed the INL (red dashed lines) and ONL (yellow dashed lines) in the central and peripheral retinas as prominent dark bands (Fig. 3*A*,*B*). The recorded INL and ONL thicknesses are plotted in Figure 3*C*. In both mice and tree shrews, the INL and ONL kept an approximately homogeneous thickness moving toward the periphery. In humans, the INL and ONL thickness significantly increased as the distance increased from the ONH (*p* = 1.0 × 10^−4^). Interestingly, the ONL, which contains photoreceptor cell bodies, appeared to be significantly thinner in tree shrews compared with the ONL in mice and humans consistently in both central and peripheral retinas (*p* = 1.0 × 10^−4^; ANOVA post hoc Tukey’s test).

### In vivo quantification of the IPL sublayers in tree shrews

**Figure 4.**
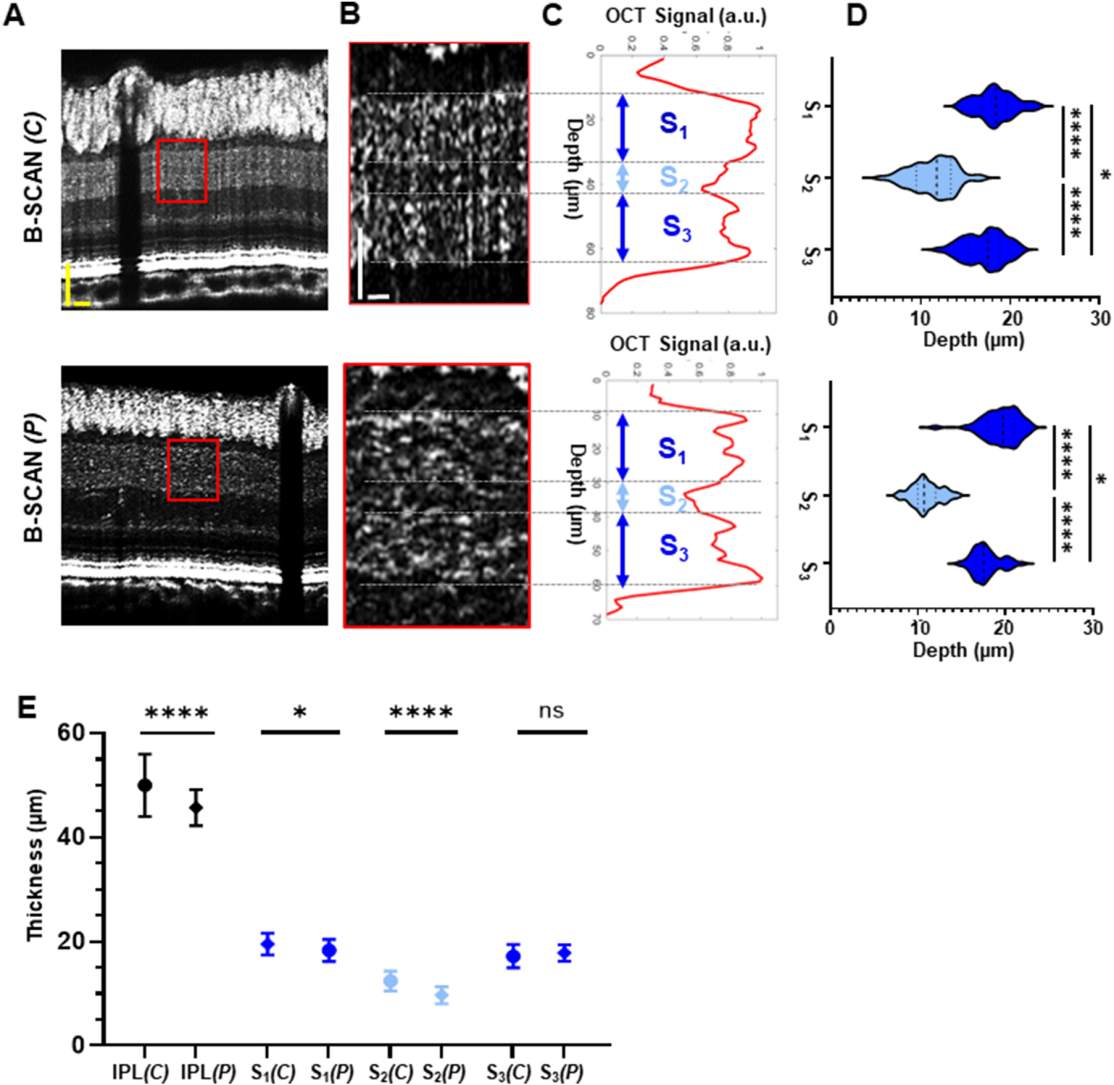
In vivo detection of IPL sublayers in tree shrews. ***A***, SR-B-scan images from central and peripheral tree shrew retinas. ***B***, Magnified view of the region highlighted by the red box in ***A*** (average of 250 SR-A-lines, corresponding to ∼545 μm along the lateral direction). ***C***, Depth-resolved A-line profiles of IPL sublayers: sublayer 1 (S_1_), sublayer 2 (S_2_), and sublayer 3 (S_3_). ***D***, Thickness quantification of S_1_, S_2_, and S_3_ in the tree shrew (*n* = 3). ***E***, Comparison of the averages of the entire IPL and individual IPL sublayers in the central and peripheral areas. IPL, inner plexiform layer; (*C*), central; (*P*), peripheral. Yellow scale bar, 50 µm; white scale bar, 25 µm. ****p < 0.0001; *p < 0.05; ns, not significant.

Taking advantage of the improved image quality offered by vis-OCT SR-B-scan and the postprocessing methods, we tested whether we could detect individual IPL sublayers in tree shrews. SR-B-scans resampled from central (500 μm radius) and peripheral (1,200 μm radius) tree shrew retinas were selected (Fig. 4*A*). The magnified view of the SR-B-scans in Figure 4*B* (red boxes) revealed two hyper-reflective bands and one hyporeflective band separating the top and bottom portions of the IPL. In the averaged A-line profiles (Fig. 4*C*), two peaks and one valley were identified as (1) sublayer S_1_ measured from the top IPL boundary to the first minimum of the valley, (2) sublayer S_2_ measured from the first minimum of the valley to the second minimum of the valley, and (3) sublayer S_3_ measured from the second minimum of the valley to the bottom IPL boundary.

Sublayer thickness quantification is reported in Figure 4*D*. S_1_ in the central region (19.5 ± 2.1; *n* = 47) was significantly thicker than S_1_ in the peripheral region (18.3 ± 2.1; *n* = 52; *p* = 3.0 × 10^−2^). Moreover, a significant thickness change was found in S_2_ the central region (12.4 ± 1.9 µm, *n* = 64) compared with that in the peripheral region (9.7 ± 1.6 µm, *n* = 49; *p* = 1.0 × 10^−4^), and no significant difference was detected in S_3_ across the retina (*C*, 17.2 ± 2.2 µm; *P*, 17.8 ± 1.5 µm; *p* = 0.63; ANOVA post hoc Tukey’s tests). Comparison of the averages of the entire IPL and the individual IPL sublayers in the central versus the peripheral area were summarized in the graph in Figure 4*E*.

### Validation of in vivo retina layer structure by confocal microscopy

**Figure 5.**
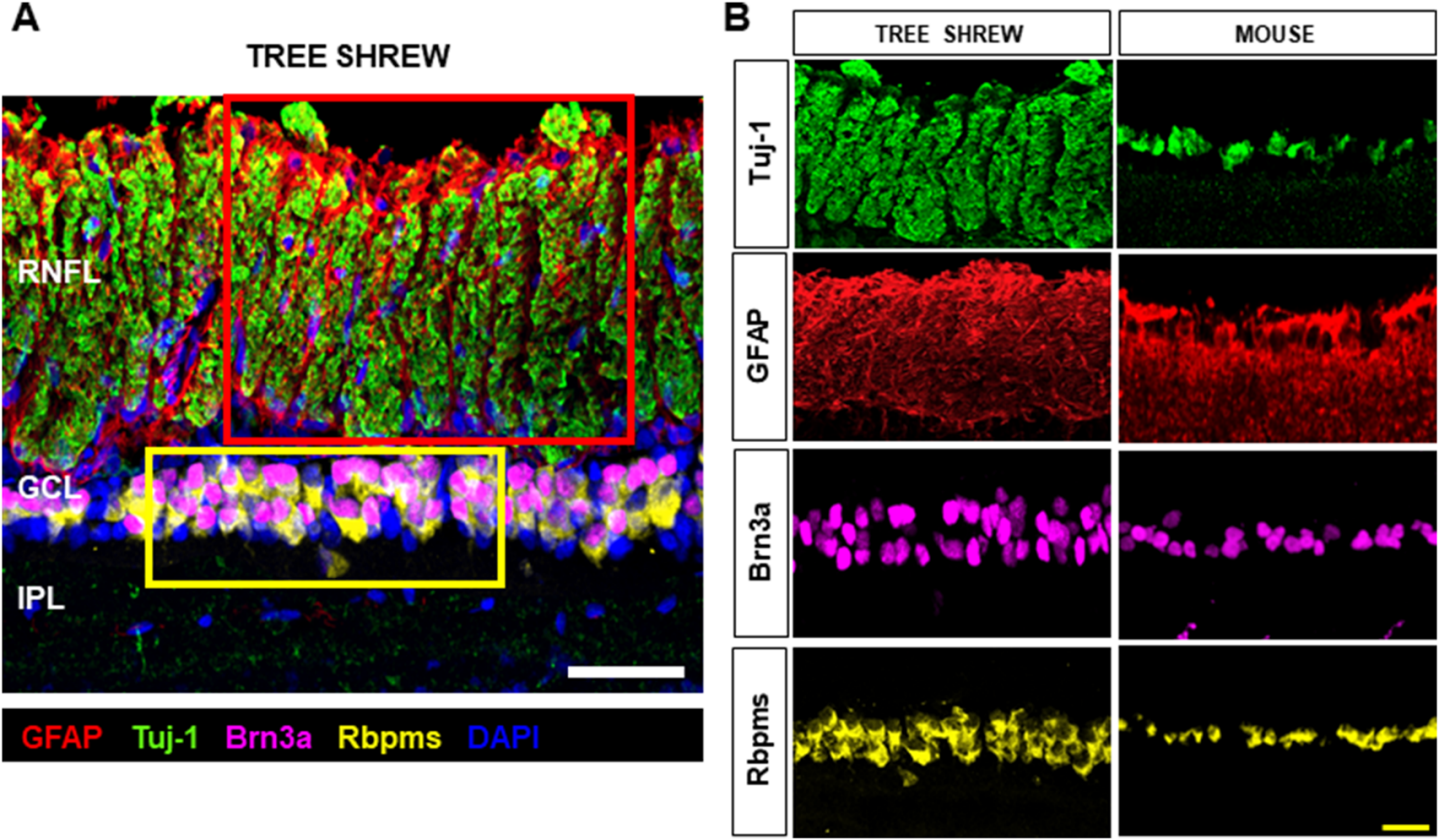
Ex vivo cross-sectional confocal images of tree shrew and mouse retinas. ***A***, Confocal cross-sectional images of the tree shrew retina immunostained with Tuj-1 (green), GFAP (red), Brn-3a (magenta), rbpms (yellow), and DAPI (blue). ***B***, The magnified view of the red box in ***A*** highlights the boundaries of individual axon bundles (Tuj-1) wrapped with astrocytes (GFAP) in tree shrews compared with the round-shaped axon bundles (Tuj-1) and astrocytes (GFAP) in mice. The magnified view of the yellow box in ***A*** reveals 3–4 soma layers of RGCs (Brn-3a, Rbpms) in the GCL in tree shews compared with 1–2 soma layers of RGCs in mice. RNFL, retinal nerve fiber layer; GCL, ganglion cell layer; IPL, inner plexiform layer. White scale bar, 50 µm; yellow scale bar, 25 µm.

A confocal image of the central tree shrew retina is shown in Figure 5*A*. The section was stained with Tuj-1 for RGC axons (green), GFAP for astrocytes/glial cells (red), Brn-3a (magenta), rbpms for RGC soma (yellow), and DAPI for cell nuclei (blue). The magnified view of the tree shrew confocal image (Fig. 5*B*) confirmed the axon bundle structures detected in vivo, with bundles wrapped by GFAP processes in the RNFL. Figure 5*B* also showed the 3–4 soma layers of RGCs in the GCL immunostained by Brn-3a and rbpms, validating the distinct GCL band captured in vivo in tree shrews (Fig. 2). In contrast, only 1–2 soma layers of RGCs were found in the mouse retina immunostained by Brn-3a and rbpms (Fig. 5*B*).

**Figure 6.**
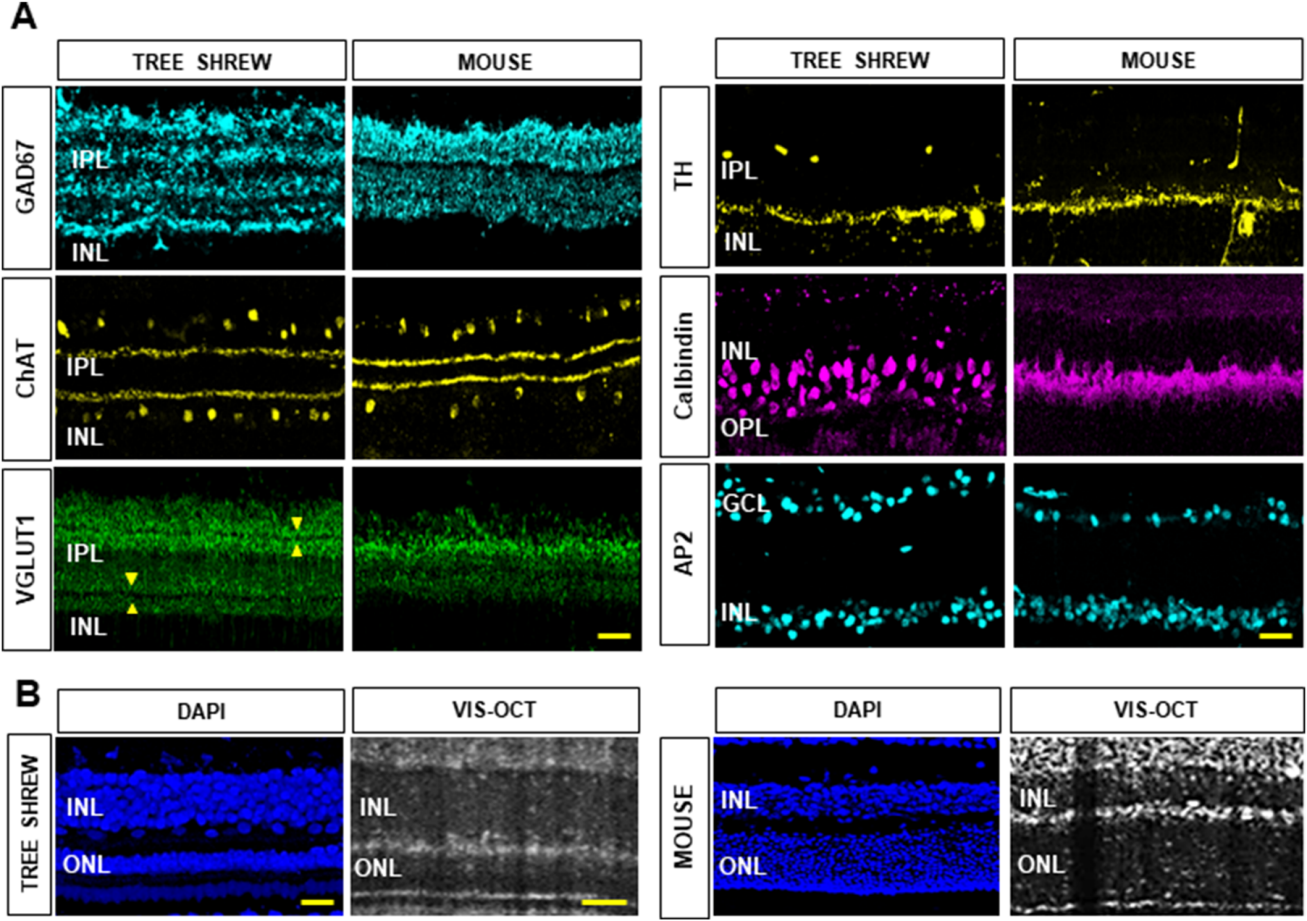
Ex vivo confocal images of the inner retina layer structure in tree shrews and mice. ***A***, Confocal microscopy images of tree shrew and mouse retina immunostained with GAD67 (light blue), ChAT (yellow), VGLUT1 (green), TH (yellow), calbindin (magenta), and AP2 (light blue). Two pairs of yellow arrowheads point to the thin dark bands in the VGLUT1 staining. ***B***, Side-by-side comparison of ex vivo confocal images stained with DAPI (blue) and in vivo SR-B-scans in a tree shrew and a mouse retina. GCL, ganglion cell layer; IPL, inner plexiform layer; INL, inner nuclear layer; ONL, outer nuclear layer. Yellow scale bars, 20 µm.

To further characterize the INL and IPL in mice and tree shrews, retinal cross sections were immunostained with glutamate decarboxylase 67 (GAD67) and ChAT antibodies to label amacrine cell (AC) somas and their processes; VGLUT1 antibody to label the bipolar cell terminals in the IPL; AP2 and TH antibodies to label GABAergic and dopaminergic ACs, respectively; calbindin antibody to label the horizontal cells and their processes; and DAPI to label nuclei in the INL and ONL. The confocal imaging of tree shrew sections (Fig. 6*A*) revealed four GAD67 sublayers, and two VGLUT1-positive layers in the IPL. Each VGLUT1-positive layer was further divided into two sublayers by a thin dark band pointed by the yellow arrowheads (bottom left panel). Overall, the ex vivo confocal images of the IPL sublayer structure confirmed the in vivo IPL findings by vis-OCT imaging. Figure 6*B* shows side-by-side comparisons of the confocal images of the INL and ONL immunostained with DAPI and the in vivo SR-B-scans of a tree shrew and a mouse retina, respectively. Confocal images also confirmed that the ONL was particularly thin in tree shrews compared with the ONL in mice as reported by the vis-OCT findings (Fig. 6*B*).

## Discussion

In this study, we presented, for the first time, a detailed, in vivo, side-by-side comparison among the tree shrew, human, and mouse retinas using vis-OCT. With improved image quality and resolution, we could detect the tree shrew IPL sublayer structures in vivo, for the first time. Additionally, we compared the thickness of the RNFL, INL, and ONL in mouse, tree shrew, and human retinas. Notably, the tree shrew model more faithfully emulates the human RNFL structure compared with the traditional rodent models, thereby holding significant promise for examining RGC loss in glaucoma or with optic nerve damage.

Tree shrews have emerged as an ideal model for studying eye diseases due to their high homology with the human eye (Albon et al., 2007; Samuels et al., 2018; Petry and Bickford, 2019; Sajdak et al., 2019). Here, we revealed that the densely packed axon bundle organization found in the tree shrew RNFL (Miller et al., 2023) is similar to the thick axon bundle network found in the human RNFL. In contrast, the mouse retina consists of a simple layer of axon bundles sparsely distributed throughout the retina (Miller et al., 2020; Grannonico et al., 2021, 2023). We further characterized the structure of the GCL and IPL in the three species. The cross-sectional SR-B-scan images revealed a distinct dark band of the GCL in tree shrews and humans versus a less defined, thin GCL in mice. The IPL, in the SR-B-scans, clearly appeared as a defined multilayered structure in tree shrews. In mice, the IPL blended with the GCL to form a single structured layer as the GCIPL. In fact, the traditional OCT imaging system often reported the overall thickness of the GCIPL due to the lack of resolution (Ruggeri et al., 2007; Huber et al., 2009; Ferguson et al., 2012; Srinivasan et al., 2014; Dysli et al., 2015). Because mice possess only a thin and rudimentary GCIPL, this limitation hinders the investigation of mechanisms by which the RGC somas and dendritic structure change with glaucoma progression.

The integrative properties of the RGC dendritic structure are critical for visual function (Sernagor et al., 2001). Since the IPL consists of various types of dendrites, a quantitative analysis of the IPL sublayer structure may provide additional information about how glaucomatous insults cause RGC damage in vivo. We showed in this study that the IPL sublayers were not detected in the mice and humans SR-B-scan due to anatomic features: the thin IPL in mice makes the in vivo separation of individual sublayers challenging (Ruggeri et al., 2007; Huber et al., 2009; Ferguson et al., 2012; Srinivasan et al., 2014; Dysli et al., 2015), and the large size of the human eye required a different vis-OCT protocol to achieve a fine resolution (Ghassabi et al., 2022). Extracting vis-OCT SR-B-scans in tree shrews allowed us to identify individual IPL sublayers relatively easily. In the IPL, we detected two hyper-reflective bands (S_1_ and S_3_) and one hyporeflective band (S_2_). S_1_ and S_2_ were significantly thicker in the central retina compared with those in the peripheral retina. S_3_ showed no significant difference between the central and peripheral retinas. RGC dendrites with ON responses terminate in the superficial portion of the IPL (S_1_ belongs to ON sublamina). In contrast, those cells with OFF responses terminate in the deep sublamina of the IPL (S_3_ belongs to OFF sublamina; Lee et al., 2016; Petry and Bickford, 2019). Thus, the complex dendrite lamination we found in the tree shrew retina is highly consistent with the functional segregation of the ON and OFF pathways investigated in primates (Grünert and Martin, 2020) and humans (Wässle, 2004; Ghassabi et al., 2022). Because of the attenuation of blood vessels in vis-OCT B-scan images as dark shadows, we speculated that the hyporeflective band (S_2_) in the IPL could represent an inner capillary plexus, not previously reported. Future investigations are required to identify and characterize the nature of the sublaminal structures.

We also found a significantly thinner ONL in the tree shrew compared with the human ONL and mouse ONL, consistent with the corresponding ex vivo confocal images. Tree shrews possess a nearly cone-exclusive (∼95%) photoreceptor retina (Kühne, 1983; Müller and Peichl, 1989; Sajdak et al., 2019), similar to primates and humans (Curcio et al., 2011; Dubra et al., 2011), while the mouse retina exhibits a preponderance of rod photoreceptors. Our results showed that rod photoreceptor outer segments appear longer in mice than those in tree shrews, highlighting a critical difference between nocturnal rodents and diurnal small mammalians.

In summary, our studies showed that vis-OCT enables the assessment of individual retinal layer structure with high axial resolution and improved contrast. Studies in human patients and animal models have demonstrated the utility of measuring the RNFL, GCL, and IPL thickness in vivo for glaucoma detection (Fujihara et al., 2020; Ghassabi et al., 2022; Mahmoudinezhad et al., 2023), and this is especially important because changes in the dendritic and axonal structure of RGCs may precede cell death (Puyang et al., 2015; Della Santina and Ou, 2017; Feng et al., 2017; Grannonico et al., 2023). The mechanisms underlying the RGC damage at the soma, dendritic, and axon levels remain controversial. Using an animal model that mimics the pathophysiology of optic neuropathies is thus essential to advance our understanding of the pathological conditions that lead to RGC death.

## Synthesis

Reviewing Editor: Silvia Pagliardini, University of Alberta Decisions are customarily a result of the Reviewing Editor and the peer reviewers coming together and discussing their recommendations until a consensus is reached. When revisions are invited, a fact-based synthesis statement explaining their decision and outlining what is needed to prepare a revision will be listed below. The following reviewer(s) agreed to reveal their identity: NONE.

The inclusion of human subjects and the comparisons to both tree shrew and mice have undeniably improved the depth of this study. By integrating human subjects, the study's findings extend beyond mere purported speculations, offering valuable practical insights with potential implications for the utility of the tree shrew as a human-like model system. Whereas, the comparative analysis with mice along only provided a nuanced understanding as it relates to the retinal structure.

With additional data from human retina imaging, the revised version provided important data to demonstrate that compared with rodent, the tree shrew retina can serve as a better, closer model for human retina studies. Especially, it is the first time the RNFL is quantified, providing significant contribution to the OCT field.

Concerns:

1. In figure 2 and 3, central(C) and peripheral(P) data are compared for each specie, yet example images are not labeled for C and P- it would be most helpful to provide corresponding example images for both C and P.

2. In line 276-277, the authors claimed "Interestingly, the ONL, which contains photoreceptor cell bodies, appeared to be considerably thinner in tree shrews compared to the ONL in mice and human", proper statistical test is needed to back up the statement.

## Author Response

The authors conducted a comparative study between Tree shrew and mouse retina, focusing mostly on the inner retina and retinal ganglion cells. The study used high-resolution OCT and confocal imaging of fixed retinal sections with immunohistochemistry. However, there are major weaknesses in the study.

The information presented mostly builds upon existing knowledge about the tree shrew retina, and there is a lack of new insights with the exception of the OCT data. The technique is novel and the in vivo assessment of RNFL and IPL layers have important clinical implication, especially the IPL sublayers revealed.

To truly elevate the impact of the study, a comprehensive reframing of the study that embraces a pure technical and/or methodological perspective as it relates to what is uncovered with respect to the OCT measurements would be helpful.

Reply: We thank the reviewers and the editor for their constructive comments. In brief, we have added imaging data from human eyes, remade figures, added antibody lists, and rewritten discussion and technical details to highlight the novelty. Please see detailed responses below.

In addition, we would like to clarify that we are concurrently preparing two manuscripts. The first manuscript has been submitted to IEEE Transactions on Medical Imaging and is available on BioRxiv 1, which was also cited in this manuscript. It focused on the technological development of vis-OCT fibergraphy (vis-OCTF) for visualizing RGC axon bundles in shrew retinal en face images. This manuscript focused on the in vivo comparative study of layer structures of retinal cross sections in mice, tree shrews, and humans using vis-OCT B-scans. There is no data overlap between the two manuscripts.

The study would be more compelling if it compared the human retina with both tree shrew and mouse retina. This would provide data to support the tree shrew as a more robust model for optic neuropathies.

Reply: We have added human retinal imaging data as suggested. We have performed vis-OCT imaging on 3 adult healthy subjects, analyzed the data and presented the retina layer structure and quantifications in the new Figure 1, Figure 2, and Figure 3 in the revised manuscript.

The limitations of the rodent model are acknowledged, but the study does not explore or quantify important differences observed, such as the abundance of rods/nuclei in the mouse retina. The fact that the tree shrew retina is predominantly composed of cones is also not discussed in detail or supported by data. Overall, the study would benefit from addressing these weaknesses to strengthen the findings.

Reply: For this study, we focused on the retinal layer structures, especially in the inner retina. First, as the reviewers pointed out, detailed studies on tree shrew cones by optics scanning ophthalmoscopy have already been published.2 By contrast, less is known about the inner retinal structures, especially the RGCs, as these cells die in glaucoma and other optic nerve neuropathy. Furthermore, we compared the overall layer structures of tree shrew's, mouse's and now human's retinas through the application of vis-OCT B-scans, which offers the possibility to resolve the features of individual layers.

Major comments:

The authors throughout the introduction and part of the discussion highlight the similarities between the tree shrew retina and human retina, however this is not really developed to any degree and would make their case a little better, perhaps if they performed a study comparing human retina vs. tree shrew vs mouse, this would create and impactful study that needs to be performed and would gain high interest in the field.

Reply: As suggested, we have added human vis-OCT data (n=3 eyes from 3 adult healthy subjects), quantified their retina layer thicknesses, and compared them with the corresponding retina layers in tree shrews and mice. Please see the new Figures 1-3 in the revised manuscript.

In the current form, there is a limited amount of new information. Many studies in the past have focused on tree shrew retina characterizing many aspects of the visual system (here is a small and incomplete list of some examples that deal with retinal characterization but there are many more: PMID: 3548881, PMID: 8713466, PMID: 9390767, PMID: 10349961, PMID: 10349961, PMID: 17394160, PMID: 2723153, PMID: 29238991). This work really doesn't add much to that realm.

Reply: First, the major novelty of the study is represented by the in vivo visualization and quantification of individual layers such as GCL and IPL, and, importantly, the IPL sublayers in tree shrews, which were not previously reported. We also compared the similarities and differences in the GCL and IPL in mice, tree shrew, and human retinas. Previous studies using traditional OCT imaging for mice and human often reported the overall GCIPL thickness due to the lack of axial resolution to identify individual layer structure 3-7. We published the first two papers to the visualization and quantification of individual axon bundles in the RNFL in mice 8, 9. We are also the first to apply vis-OCT B-scans to visualize individual retinal layers in tree shrews as described in this manuscript. In our other manuscript 1, we developed vis-OCTF to visualize RNFL and axon bundle patterns en face, which does not overlap with we presented in this study.

Minor concerns:

1. Why did you use AP-2 as an inner retinal marker, why not Parvalbumin (PV), and others.

Reply: We have added three antibody tables in the revised manuscript. We indeed tested one PV antibody in both mouse and tree shrew, but unfortunately the one we tested did not work well. Below is the supplemental table which is a list of all the primary antibodies that were tested in this study.

Supplemental Table I: List of all the primary antibodies tested in tree shrew and mouse retinas. [C]: concentration. TS: tree shrew; MS: mouse; Y: yes; N: no.

2. Figure 3: the multiple layering of the GCL is likely due to the oblique nature of cryosectioning, normally in the rodent the ganglion cell layer is consistently a single layer, and I believe the same is true for the Tree shrew. So that would likely explain the multiple cell layers observed.

Reply: We respectfully disagree with the reviewer's comment. In mice, the GCL is a thin layer characterized by one or two soma layers of RGCs, as shown by the electron microscopy (EM) picture of the mouse retina (below)10. The red box highlights GCL. In addition, on average each mouse retina only contains about 40-60k RGCs, so their axon bundles form a thin loose layer, not densely packed as seen in tree shrew or human retinas (see the new Figure 1).

In addition, other studies confirmed that tree shrew11, primates12, and human12 GCL presents multiple layers of RGC somas as shown below. (Left panel) Electron microscopy picture of tree shrew retina section11. (Middle panel) Light micrographs of vertical sections through central macaque retina taken in central12. (Right panel) Confocal image of a vertical section through the center of the fovea of a human retina12. Red boxes highlight GCL.

3. Figure 4B, in the mouse GA67 labeling it appears that you only see 2 layers or sublamina, the ON and OFF, in the image shown, there is no third sublamina? Reply: Thank you for the clarification. We deleted the sentence in the revised manuscript.

4.Why didn't the authors label the retinas with VGLUT1? This clearly shows the ON and OFF sublamina of the inner retina.

Reply: Thank you for the suggestion. We added VGLUT1 stained in tree shrew and mouse sections (new Figure 6) in the revised manuscript.

RBPMS has been shown to be a superior marker to Brn3a of retinal ganglion cells in most mammalian species (PMID: 24318667), I would stick with that and not bother with Brn3a and others. It labels all the RGCs! Reply: We used both antibodies (RBPMS and Brn3a) because the results will validate each other and our long-term goal in future studies is to examine subtype-RGCs specific changes in optic neuropathies.

The figure 5 highlights the IPL sublayers, however Fig 5D THE PERIPHERAL VS CENTRAL are not directly compared, and based on Fig4C TS c vs. TS p are significantly different? But based on reported numbers in text and 5E, they are not significantly different. 5E should be turned into a bar graph, and statistics should be performed for central vs. peripheral s there any significance? Reply: As suggested, we carefully reexamined the data, changed Figure 5E into a graph, and performed a statistical analysis of the central versus peripheral area in the revised manuscript.

Minor issues Line 55. RGCs is >10X lower --> less than 10%.

Reply: Thank you for the suggestion. We changed it.

Line 332-222 citations are needed for the statement.

Reply: We added several references (line 235) Line 335 the lack of knowledge should be specified.

Reply: We rephrased the sentence.

Miller 2020a and Miller 2020b are the same.

Reply: Thank you. We fixed it.- REFERENCES 1. Miller DA, Grannonico M, Liu M, Savier E, McHaney K, Erisir A, Netland PA, Cang J, Liu X, Zhang HF. Visible-Light Optical Coherence Tomography Fibergraphy of the Tree Shrew Retinal Ganglion Cell Axon Bundles. bioRxiv. 2023. Epub 2023/06/09. doi: 10.1101/2023.05.16.541062. PubMed PMID: 37293064; PMCID: PMC10245691.

2. Sajdak BS, Salmon AE, Cava JA, Allen KP, Freling S, Ramamirtham R, Norton TT, Roorda A, Carroll J. Noninvasive imaging of the tree shrew eye: Wavefront analysis and retinal imaging with correlative histology. Exp Eye Res. 2019;185:107683. Epub 2019/06/04. doi: 10.1016/j.exer.2019.05.023. PubMed PMID: 31158381; PMCID: PMC6698412.

3. Srinivasan PP, Heflin SJ, Izatt JA, Arshavsky VY, Farsiu S. Automatic segmentation of up to ten layer boundaries in SD-OCT images of the mouse retina with and without missing layers due to pathology. Biomed Opt Express. 2014;5(2):348-65. doi: 10.1364/BOE.5.000348.

4. Ruggeri M, Wehbe H, Jiao S, Gregori G, Jockovich ME, Hackam A, Duan Y, Puliafito CA. In Vivo Three-Dimensional High-Resolution Imaging of Rodent Retina with Spectral-Domain Optical Coherence Tomography. Investigative ophthalmology & visual science. 2007;48(4):1808-14. doi: 10.1167/iovs.06-0815.

5. Dysli C, Enzmann V, Sznitman R, Zinkernagel MS. Quantitative Analysis of Mouse Retinal Layers Using Automated Segmentation of Spectral Domain Optical Coherence Tomography Images. Translational vision science & technology. 2015;4(4):9-. doi: 10.1167/tvst.4.4.9. PubMed PMID: 26336634.

6. Huber G, Beck SC, Grimm C, Sahaboglu-Tekgoz A, Paquet-Durand F, Wenzel A, Humphries P, Redmond TM, Seeliger MW, Fischer MD. Spectral Domain Optical Coherence Tomography in Mouse Models of Retinal Degeneration. Investigative ophthalmology & visual science. 2009;50(12):5888-95. doi: 10.1167/iovs.09-3724.

7. Ferguson LR, Balaiya S, Grover S, Chalam KV. Modified protocol for in vivo imaging of wild-type mouse retina with customized miniature spectral domain optical coherence tomography (SD-OCT) device. Biological Procedures Online. 2012;14(1):9. doi: 10.1186/1480-9222-14-9.

8. Grannonico M, Miller DA, Liu M, Norat P, Deppmann CD, Netland PA, Zhang HF, Liu X. Global and Regional Damages in Retinal Ganglion Cell Axon Bundles Monitored Non-Invasively by Visible-Light Optical Coherence Tomography Fibergraphy. J Neurosci. 2021;41(49):10179-93. Epub 2021/10/28. doi: 10.1523/JNEUROSCI.0844-21.2021. PubMed PMID: 34702745; PMCID: PMC8660041.

9. Miller DA, Grannonico M, Liu M, Kuranov RV, Netland PA, Liu X, Zhang HF. Visible-Light Optical Coherence Tomography Fibergraphy for Quantitative Imaging of Retinal Ganglion Cell Axon Bundles. Transl Vis Sci Technol. 2020;9(11):11. Epub 2020/10/29. doi: 10.1167/tvst.9.11.11. PubMed PMID: 33110707; PMCID: PMC7552935.

10. Alex AF, Alnawaiseh M, Heiduschka P, Eter N. Retinal Fundus Imaging in Mouse Models of Retinal Diseases. In: Weber BHF, Langmann T, editors. Retinal Degeneration: Methods and Protocols. New York, NY: Springer New York; 2019. p. 253-83.

11. Abbott CJ, McBrien NA, Grünert U, Pianta MJ. Relationship of the Optical Coherence Tomography Signal to Underlying Retinal Histology in the Tree Shrew (Tupaia belangeri). Investigative ophthalmology & visual science. 2009;50(1):414-23. doi: 10.1167/iovs.07-1197.

12. Grünert U, Martin PR. Cell types and cell circuits in human and non-human primate retina. Prog Retin Eye Res. 2020;78:100844. Epub 2020/02/08. doi: 10.1016/j.preteyeres.2020.100844. PubMed PMID: 32032773.
